# Emergency treatment of complicated incisional hernias: a case study

**DOI:** 10.1186/1750-1164-3-15

**Published:** 2009-12-17

**Authors:** Francesco La Mura, Roberto Cirocchi, Eriberto Farinella, Umberto Morelli, Vincenzo Napolitano, Lorenzo Cattorini, Alessandro Spizzirri, Barbara Rossetti, Pamela Delmonaco, Carla Migliaccio, Diego Milani, Piero Covarelli, Carlo Boselli, Giuseppe Noya, Francesco Sciannameo

**Affiliations:** 1 General Surgical Unit, St. Maria Hospital, Terni (TR), University of Perugia, Italy

## Abstract

**Background:**

The emergency treatment of incisional hernias is infrequent but it can be complicated with strangulation or obstruction and in some cases the surgical approach may also include an intestinal resection with the possibility of peritoneal contamination. Our study aims at reporting our experience in the emergency treatment of complicated incisional hernias.

**Methods:**

Since January 1999 till July 2008, 89 patients (55 males and 34 females) were treated for complicated incisional hernias in emergency. The patients were divided in two groups: Group I consisting of 33 patients that were treated with prosthesis apposition and Group II, consisting of 56 patients that were treated by performing a direct abdominal wall muscles suture.

**Results:**

All the patients underwent a 6-month follow up; we noticed 9 recurrences (9/56, 16%) in the patients treated with direct abdominal wall muscles suture and 1 recurrence (1/33, 3%) in the group of patients treated with the prosthesis apposition.

**Conclusions:**

According to our experience, the emergency treatment of complicated incisional hernias through prosthesis apposition is always feasible and ensures less post-operative complications (16% vs 21,2%) and recurrences (3% vs 16%) compared to the patients treated with direct muscular suture.

## Background

Repair of abdominal wall hernia represents the most common group of operatios performed by general surgeons all around the world. Incisional hernia is a serious complication after abdominal surgery which occurs in 11-23% of laparotomies [1].

In 2003 it was estimated that over 100,000 ventral incisional hernia repairs were performed in the US. Risk factors for incisional hernia formation and preventive strategies are not clearly defined, but according to data from literature, significant demographic factors influencing incisional hernia incidence are age (> 45 years) and male gender. Preoperative anaemia (Hb < 100 g/l) and BMI > 25, associated with previous laparotomies and postoperative catecholamin-therapy also seem to play an important role [2]. The tension-free repair is one of the key concepts in hernia surgery. The use of a mesh prosthesis decreases the recurrence rates, particularly for inguinal and incisional hernias. Recently, the laparoscopic approach extended the options and approaches for repairing the fascial defect.

The emergency treatment of incisional hernias is not frequent [3], and its technical approach can be different from the elective one, both for the septic conditions in which the emergency treatment is usually performed and for the patients' age which can lead to several technical difficulties. As an emergency, it often occurs in elderly patients with voluminous hernias complicated with strangulation or obstruction [4]. In some of these cases the surgical approach may also include an intestinal resection, with the possibility of peritoneal contamination [5]. This study aims at reporting our experience in the emergency treatment of complicated incisional hernias, analysing the results obtained with the employment of synthetic prosthesis versus the open surgical repair.

## Materials and methods

We performed a clinical study by revising clinical notes, through which we evaluated the different treatments of patients with complicated incisional hernias. All the patients whose hernia were only an attendant pathology and did not represent itself the cause for an emergency surgical treatment, were excluded fom the trial.

Since January 2001 till July 2008, 89 patients (55 males and 34 females) were treated for complicated incisional hernias in emergency. We divided the patients in two different groups. The patients treated with prosthesis apposition (Group I) were 33 (24 males and 9 females) while 56 patients (Group II) (31 males and 25 females) were treated by performing a direct abdominal wall muscles suture. Five of these patients (8,9%) had such voluminous incisional hernias that they could not be treated by carrying out a direct abdominal wall muscles suture (Table 1).

**Table 1:** Emergency treatment of complicated incisional hernias

	Prosthesis apposition (Group I)	Direct abdominal wall muscles suture (Group II)
**Patients treated**	33	56

**Omentum resections**	14	41

**Intestinal resections**	6	15

## Results

As concerns the group treated with the apposition of prosthesis (Group I), obstruction with no possibility of reduction occurred in 27 cases; in these cases we performed an adhesiolisis and a greater omentum resection (14 patients) which eased the abdominal replacement of the ileal ansae, being the omentum often inflamed, thickened and fibrous. In the remaining 6 cases, strangulation was the reason for an emergency treatment; in these cases we performed a resection of the necrotic ileum and the intestinal continuity was restored by carrying out a mechanic suture. Strangulation occurred in those patients whose incisional hernias had a narrow neck. We registered 7 complications; 4 parcellar cutaneous necrosis, 2 hematomas and 1 wound suppuration (Table 2).

**Table 2:** Complications

	Prosthesis apposition (Group I)	Direct abdominal wall muscles suture (Group II)
**Total complications**	7 (21%)	24 (43%)

**Parcellar cutaneous necrosis**	4 (57%)	7 (29%)

**Hematomas**	2 (29%)	6 (25%)

**Wound suppuration**	1 (14%)	11 (46%)

The surgical technique performed for Group I patients, was carried out through the apposition of polypropylene prosthesis which was placed between the posterior rectus wall and the anterior wall of the rectus sheath. The prosthesis was fixed by non-absorbable interrupted stitches which were sequentially placed through the aponeurosis, the prosthetis, back into the prosthetis and finally again through the aponeurosis, approximately 0.5 cm by the entrance point. The suture must be performed at least 2 cm far from the hernia edge, in a totally sane tissue. On the contrary the patients with voluminous incisional hernias were treated with a PTFE mesh (polytetrafluoroethylene) which was placed intraperitoneally, in contact with the abdominal viscera. The employment of PTFE allows the reduction of viscero-parietal adhesions and the constitution of a stronger abdominal wall.

In the patients treated with a direct abdominal wall muscles suture (Group II) we carried out an adhesiolysis and a greater omentum resection in 41 patients. In 15 cases we performed a resection of the necrotic ileum followed by a mechanic suture. We registered 24 complications; 7 parcellar cutaneous necrosis, 6 hematomas and 11 wound suppurations (Table 2).

All the patients underwent a 6-month follow up; we noticed 9 recurrences (9/56, 16%) in the patients treated with direct abdominal wall muscles suture and 1 recurrence (1/33, 3%) in the group of patients treated with the prostesis apposition (Table 3).

**Table 3:** Recurrences

	Prosthesis apposition (Group I)	Direct abdominal wall muscles suture (Group II)
**Recurrences**	1 (3%)	9 (16%)

## Discussion

Incisional hernias can range in size from very small to large and complex ones and appear as a bulge by the area of a previous surgical scar. Nearly any prior abdominal operation can develop an incisional hernia, however the most frequent site is along incisions running down from the breastbone to the pubic area. These hernias may occur after large surgeries such as intestinal or vascular surgery, but also after an appendectomy or even through the small scar of a laparoscopy wound. Surgical repair of incisional hernias is usually recommended, as they can become a medical or surgical emergency.

An incisional hernia can be defined as complicated when the involved structures undergo worsening conditions. Particularly, the concerned structures may be described as follows:

1) Cutaneous: large and thin scars, cutaneous atrophy and eczemas, suppurative flogosis, fistulae.

2) Hernial sac: multiple sacs, fibrous septa, sac thickening and adhesions.

3) Visceral: chronic incarceration, obstruction, strangulation, ileum and colonic torsion with progressive damage up to gangrene; greater omentum involvement, mesenteritis and perivisceritis.

The frequency of complicated incisional hernias varies from 10 to 40%. The most frequent complications are incarceration, obstruction and strangulation [6].

There are two main factors for the pathogeneses of these complications: the hernial orifice rigidity and the presence of tenacious adhesions between the hernial sac and its content or between the sac and the surrounding tissues.

The formation of viscero-visceral and viscero-parietal bridles is the necessary condition for the production of strangulation: the intestinal loop contained into the sac is firstly affected by a transit alteration and later on by circulatory disturbance.

The strangulated intestinal tract rapidly goes towards congestion, edema and turgor caused by a disturbance of venous circulation which is followed by the formation of trasudate into the intestinal loop entrapped into the hernial sac; the intestinal wall goes towards progressive modifications up to necrosis and perforation. The omentum can be involved in the strangulation process; in such case the affected part adheres to the hernial sac and turns into a fibrous tissue. In case of strangulation, the symptoms will be those of a typical intestinal occlusion or subocclusion, depending on the elapsed time and the single material cause, although the synthomatology can sometimes be hard to define; for instance, in obese abdomens it is not easy to recognize the strangulation of small laparoceles.

The emergency surgical treatment for complicated incisional hernias, besides the problems given by the lack of intestinal preparation, shows a higher morbidity also due to the development of an acute respiratory failure; this is caused by the abdominal reduction of the herniated viscera which causes an increase of endoabdominal pressure pushing up the diaphragm [7].

Since the newly formed subcutaneous cavity resulting from the hernia reduction can be origin of haematic collections formation associated with a frequent necrosis of the cutaneous rims, we suggest to perform a cutaneous and subcutaneous resection in order to avoid both complications. Furthermore in all the patients we normally place one or two subcutaneous Jackson-Pratt suction drainages.

At the moment the most frequent treatment of voluminous incisional hernias is performed through the use of synthetic prosthesis, which allows the abdominal wall reconstruction according to the "tension free" technique.

The prosthesis is to be placed between the peritoneum and the posterior surface of the rectus abdominis muscles [8,9] or between the posterior surface of the rectus abdominis muscles and their posterior sheath [10]. In both cases the prosthesis apposition allows a strength discharge upon the abdominal wall circumference; moreover, the overlap of the muscle to the prosthesis (properitoneal technique) allows a wider distribution surface of tension strengths. In order to avoid a prosthesis dislocation, it is necessary to fix it to the abdominal wall by non-absorbable interrupted stitches (prolene). The prosthesis must be considerably wider than the parietal breach, so that the endoabdominal pressure might ease its adhesion to the abdominal wall. The anterior rectus muscles sheath is closed by a continuous non-absorbable suture. When it is not possible to suture the abdominal wall because of a massive tissutal loss, we place a PTFE prosthesis in contact with the viscera and we suture it to the muscle sheaths [11,12]. PTFE avoids visceral adhesions, assuring this material to be used in properitoneal locations for voluminous incisional hernias when it is not possible to perform a direct peritoneal suture; it also lowers the infections incidence and causes only a weak foreign body reaction [13-19] (Appendix 1).

The described techniques performed in emergency do not show a higher incidence of complications (fistulisation, hematomas and wound dehiscence) compared to elective surgery [20,21]; also according to our experience, the emergency treatment of complicated incisional hernias appears to be feasible, both in terms of post-operative complications and recurrences.

## Conclusions

According to our experience, the emergency treatment of complicated incisional hernias through prosthesis apposition is always feasible and ensures less post-operative complications (21% vs 43%) and recurrences (3% vs 16%) compared to the patients treated with direct muscular suture.

The technical approach in emergency is correct when considering the clinical conditions of the patient, the size of the hernia and each eventual complication; such a dealing will minimize the complication rate and make it comparable to the one achieved with elective practice. Furthermore, synthetic prosthesis allows defects of any size to be repaired without tension and with a lower recurrence rate, providing an added support to the weak abdominal wall.

## Competing interests

The authors declare that they have no competing interests.

## Consent section

All the patients were informed that their clinical history would be used for a study. All of them signed a personal data treatment consent. No image was used.

## Authors' contributions

FL cooperated in writing the article and translated it into English

RC drafted the article

EF checked the numbers and percentages

UM updated the references

VN made the tables

LC formatted the article

AS searched for the references

BR collected patients' data

PD chose the most useful and interesting articles in literature about the field

CM searched for the references

DM collected the patients' consent

PC gave some language suggestions

CB formatted the references

GN supervised the article production

FS allowed the collection of the patients' data and supervised the whole work making

All the authors read and approved the final version of the manuscript

## Appendix 1

Features of PTFE prosthesis:

Low infections incidence

Absorbability by connective tissues

Low incidence of adhesions

Weak foreign body reaction
